# Analysis of the genomes and phylogenetic relationships of lytic *Pseudomonas aeruginosa* PA14 phages BL5, BL8, and BL9 from aquatic sources

**DOI:** 10.1128/mra.00659-25

**Published:** 2025-09-12

**Authors:** Fahareen B. Mosharraf, Enoch Ghosh, Carson Bellew, Austen Rowell, Lisa M. Bono

**Affiliations:** 1 Department of Biological Sciences, Texas Tech University 124573 , Lubbock, Texas, USA; DOE Joint Genome Institute, Berkeley, California, USA

**Keywords:** bacteriophages, environmental isolates, genomic characterization, phylogenetic analysis

## Abstract

Three lytic bacteriophages targeting *Pseudomonas aeruginosa* PA14 were isolated from Lubbock, TX, playa lakes. Genomic sequencing and bioinformatic analysis classified these phages as unclassified species in the *Pbunavirus* genus of the *Caudoviricetes* class.

## ANNOUNCEMENT

*Pseudomonas aeruginosa* PA14 is a standard laboratory strain and a highly drug-resistant, Gram-negative opportunistic pathogen commonly found in environments, such as soil and water (1–4). We report the isolation, sequencing, and characterization of three bacteriophages infecting PA14, sourced from playa lakes (ephemeral, rain-filled ponds and wetlands) in Lubbock, Texas.

On 11 August 2022, water samples were collected from Lubbock, TX, at depths of 7–15 cm, at coordinates 33.566°, –101.803° (BL5 and BL8) and 33.511°, –102.002° (BL9). Samples were filtered successively through 0.45-micron and 0.22-micron syringe filters to eliminate debris and non-intended microbial cells, respectively. Each filtered sample (200 µL) was mixed with 400 µL of stationary PA14 culture and incubated at 37°C overnight. Post-incubation, samples were centrifuged at 1,786×*g* for 30 min and filtered again through a 0.22-micron filter. Phages were isolated via the agar-overlay method on PA14 lawns (5), followed by triple plaque purification. Phage DNA was extracted using the Quantabio Extraction DNA Kit, with quality and concentration assessed via the BioTek Take3 microvolume plate. DNA libraries were prepared with the Illumina DNA Prep Tagmentation Kit and sequenced on the Illumina NextSeq 2000 using a 300-cycle flow cell kit, producing 150-bp paired-end reads.

Raw sequence data were assembled, and the genome was annotated, following our earlier announcements (6, 7). The software fastp v0.23.4 (8) was used for trimming and quality control. Host contaminants (NCBI GeneBank accession no. NZ_CP127126.1) were removed using bowtie2 (9). Genomes were assembled with SPAdes v3.15.5 (10), and coverage and depth were evaluated using SAMtools v1.6 (11) and BEDTools v2.31.0 (12). Assembly metrics were generated with Quast v2.2.4 (13), and genome completeness was verified using CheckV v1.0.1 (14). Taxonomic classification was performed with NCBI BLASTn, aligning sequences to the NCBI nucleotide database. Genomes were annotated using PHROGS via Pharokka v1.3.2 (15, 16). We assessed the sequences for alternative codon usage using both Pharokka Prodigal algorithm (17) and NCBI ORF finder with standard genetic code and alternative start/stop codon usage option. FastANI v1.34 (18) was used for pairwise genome comparisons to identify closest relatives. All software was used with default settings. Phage morphology was examined using a Hitachi H-7650 TEM at TTU’s CASM facility, with 1% uranyl acetate staining.

**Fig 1:**
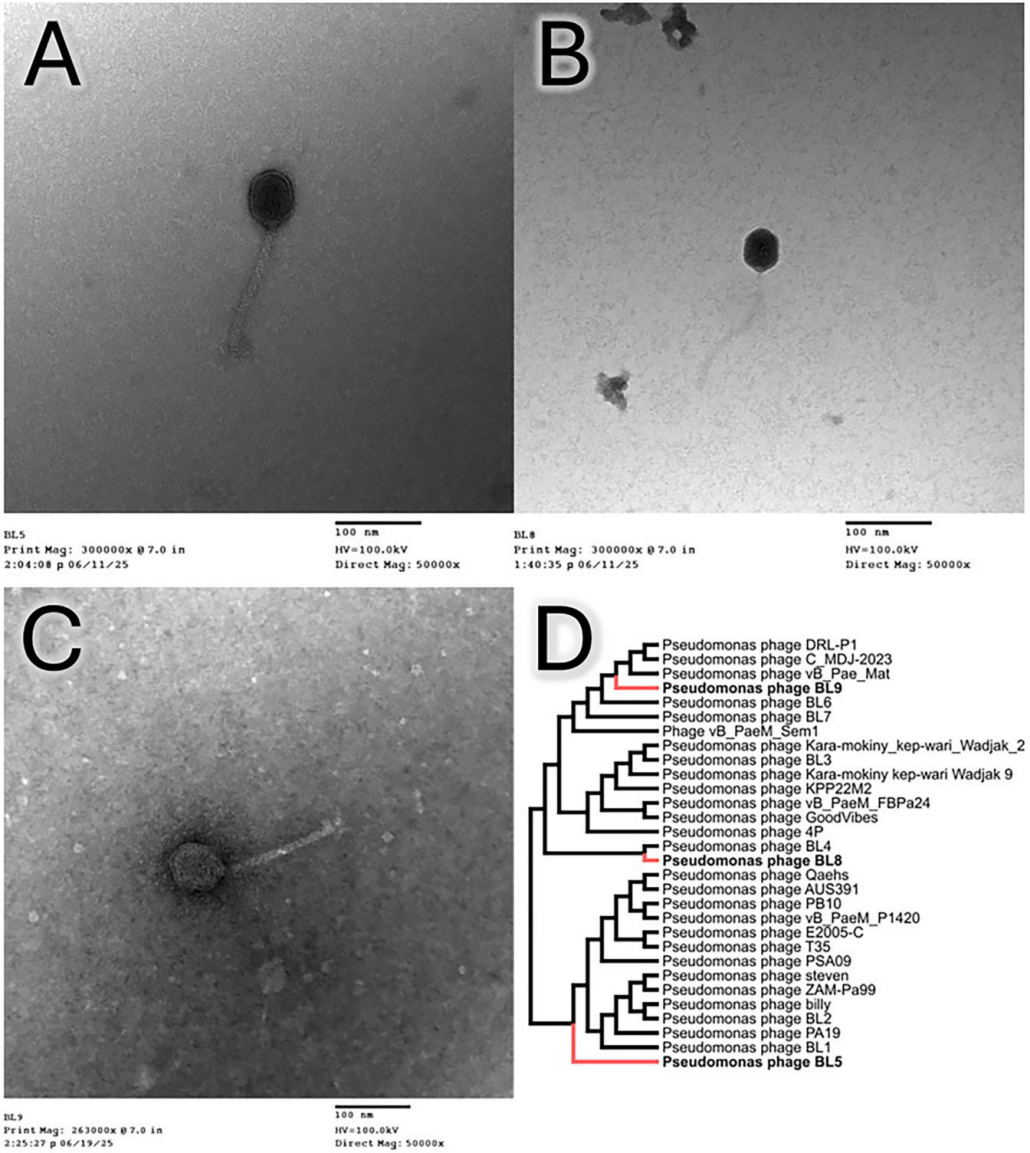
Bacteriophage micrographs and phylogenetic tree. (**A–C**) Electron micrographs of tailed bacteriophages BL5, BL8, and BL9, respectively. Average total length of 160 nm and head diameter of 50 nm. (**D**) Cladogram depicting relationship of isolated phages (in bold and with red branches) to classified Pbunavirus sequences from NCBI nucleotide database, including other BL phages isolated from playa lakes in Lubbock, TX, in August 2022.

Assembly results showed the existence of a single contig >5,000 bp. Using all sequence annotation tools, we confirmed consistent absence of stop codon for BL5; consequently, the genome sequence for BL5 was categorized as partial; however, BL8 and BL9 were categorized as complete. Sequence analysis (see Table 1) placed the phages in an unclassified species within the genus *Pbunavirus* in the class *Caudoviricetes*. Despite being classified similarly, these phages do not share the same host as those identified from earlier work (6, 7). Electron microscopy showed phage particles with lengths of 160 nm and head diameters of 50 nm (Fig. 1A through C). Whole genome sequences from the NCBI database (retrieved 12 June 2025) (19) were aligned with MAFFT v7.526 (20), and phylogenetic trees were inferred using IQ-TREE v2.3.6 (21), visualized with FigTree v1.4.4 (22) (Fig. 1D).

**TABLE 1:** Characterization of the reported bacteriophage BL5, BL8, and BL9

Parameter	BL5	BL8	BL9
Genome size (bp)	58,933	61,426	65,757
Total no. of reads generated per sample	663,962	53,526	777,082
Average coverage	1,068	925	1,378
CheckV completeness (%)	100%	100%	100%
CheckV quality	High	High	High
GC content (%)	59.17%	59.22%	54.90%
No. of coding sequence (CDs)	89	88	36
No. of hypothetical proteins	45	53	159
No. of tRNAs	0	0	0
Virulence genes	0	0	0
Antibiotic resistance genes	0	0	0
Average nucleotide identity (ANI values in %)	92.32%	98.11%	97.97%
Closest related phage based on average nucleotide identity (GenBank accession no.)	*Pseudomonas* phage vB_PaeS_PAJD-1 NC_072500	*Pseudomonas* phage Chuck OQ992557.1	*Pseudomonas* phage EPa61 NC_048744.1
GeneBank accession no.	PP791527	PQ474299	PQ474300
Bioproject accession no.	PRJNA1043699	PRJNA1173231	PRJNA1173232
SRA accession no.	SRP479098	SRP590222	SRP590223

## Data Availability

Phage genomes were deposited in the GenBank, Bioproject, and NCBI Sequence Read Archive databases (SRA) and the accession numbers are listed in Table 1.
